# “Claw hand with a unilateral onset” as a regional variant of Guillain-Barre’ syndrome: A case report

**DOI:** 10.1097/MD.0000000000020227

**Published:** 2020-05-15

**Authors:** Suhong Wang, Shuxin Zhao, Zhecheng Zhang

**Affiliations:** a Department of Neurology, Third Central Hospital of Tianjin; b Tianjin Institute of Hepatobiliary Disease; c Tianjin Key Laboratory of Artificial Cells; d Artificial Cell Engineering Technology Research Center of Public Health Ministry; e Department of Endocrinology, Third Central Hospital of Tianjin, Tianjin, China.

**Keywords:** Claw hand, Guillain-Barre’ syndrome, regional variant of Guillain-Barre’ syndrome

## Abstract

**Rationale:**

Although distal nerves located at sites prone to compression are susceptible to autoimmune attack, Guillain-Barre’ syndrome (GBS) with exclusive hand muscle involvement is rarely found in clinics. All reported patients presented with a special variant - finger extensor weakness, especially claw hand caused by predominant ulnar extensor involvement. Similar to typical GBS, these patients showed bilateral symmetric onset with rapid clinical progression.

**Patient concerns:**

A 62-year-old man with GBS was admitted to our hospital with unilateral onset of claw hand. He showed relatively slow progression and did not develop bilateral symmetric claw hands until 6 weeks later.

**Diagnoses:**

Eventually the patient was diagnosed as having a regional variant of GBS by neuronal electrophysiology and cerebrospinal fluid examinations.

**Interventions:**

This patient was treated with intravenous thrombolysis within 4.5 hours of onset. Eventually he was diagnosed as having a regional variant of GBS and was treated with gamma-globulin (400 mg/kg/d) for 5 consecutive days via intravenous infusion.

**Outcomes:**

The patient had a slow recovery with persistent mild finger extensor weakness.

**Lessons:**

This patient presented with unilateral onset of claw hand, and the diagnosis of acute ischemic stroke could not be excluded because of a short time window; hence, he was treated with intravenous thrombolysis within 4.5 hours of onset. Eventually he was diagnosed as having a regional variant of GBS. It is important that GBS should also be considered in patients with unilateral hand weakness and unknown aetiology in the early stages of disease.

## 1 Introduction

As cases of localized Guillain-Barre’ syndrome (GBS) with various manifestations are continuously being reported, the disease spectrum of GBS continues to expand. Although distal nerves located at sites prone to compression are susceptible to autoimmune attack, GBS with exclusive hand muscle involvement is rarely found in clinics. In the reported cases,^[1,2,3,4,5,6,7]^ patients presented with finger extensor weakness, especially claw hand caused by predominant ulnar extensor involvement. Similar to typical GBS, all of the patients showed bilateral symmetric onset and rapid clinical progression. Here we describe an uncommon case of GBS with claw hand and unilateral onset. The patient showed relatively slow progression and did not develop bilateral symmetric claw hands until 6 weeks later. Our case is reported below, and relevant publications are reviewed.

## 2 Case report

**Figure 1:**
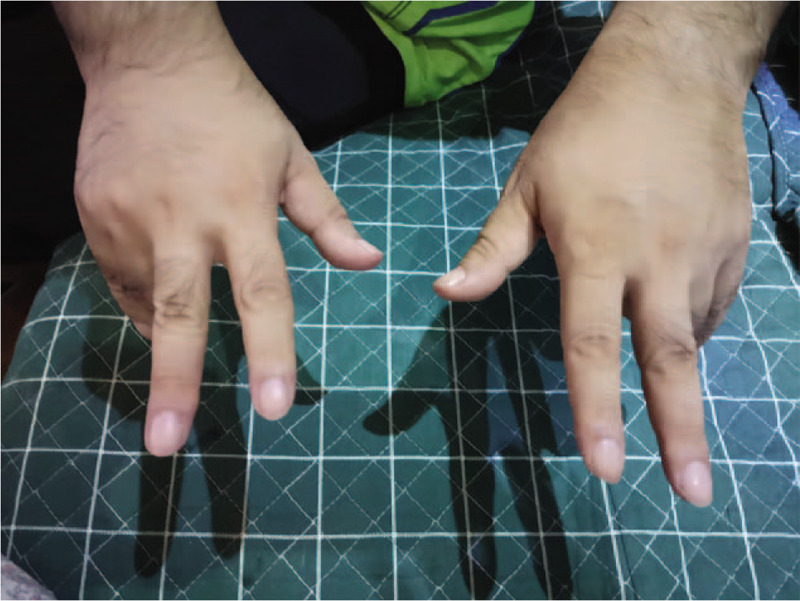
Bilateral claw hand. The patient showed significantly decreased muscle strength in the finger ulnar extensors; and the strength of finger flexion, wrist extension and wrist flexion was normal.

A 62-year-old man was admitted to our hospital with acute onset of right-sided claw hand accompanied by mild numbness. The patient had a history of hypertension but no prior history of infection. Physical examination revealed significantly decreased muscle strength with power of a 3/5 grade in the abductor digiti minimi, and ulnar lumbrical and interosseus muscles of the right hand; however, the strength of finger flexion, wrist extension and wrist flexion was normal. The patient had a normal level of consciousness and speech, and normal cranial nerves and bilateral lower limb strength. The tendon reflexes of the limbs were absent, and Babinski sign was negative. No abnormalities were found on the laboratory examination, neuronal electrophysiology, computed tomography, and magnetic resonance imaging of the brain and cervical spine. The diagnosis of acute ischemic stroke could not be excluded in this patient based on the clinical manifestations; hence, he was treated with intravenous thrombolysis within 4.5 hours of onset. However, he did not show any significant improvement. The patient's unilateral symptoms remained stable until 6 weeks later, when he developed bilateral symmetric claw hands (Fig. 1). Neuronal electrophysiology during re-examination revealed significantly reduced motor and sensory fiber action potential amplitudes of bilateral ulnar and radial nerves (Table 1), a small amount of denervated potential of the abductor digiti minimi and extensor digitorum communis on needle electromyography, and absence of conduction block or F-wave abnormalities. Cerebrospinal fluid examination indicated albuminocytologic dissociation and positive antiganglioside GM_1_ antibody. The patient was diagnosed as having a regional variant of GBS, and his symptoms were gradually alleviated after administration of gamma-globulin (400 mg/kg/d) for 5 consecutive days via intravenous infusion. After 1 year of follow-up, the patient still had mild weakness of bilateral hand extensors.

**Table 1:**
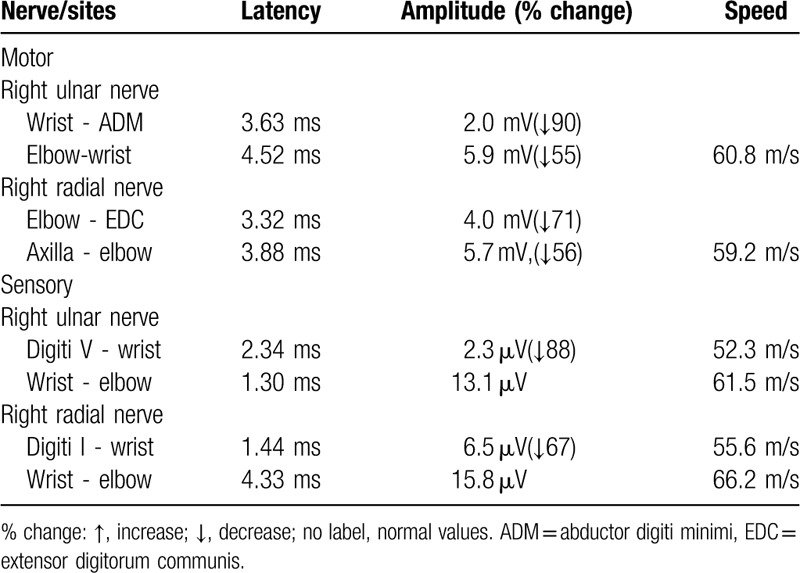
Result of ulnar and radial nerve conduction study.

## 3 Discussion

GBS with exclusive hand muscle involvement is uncommon. In patients with this GBS type, the hands present a special variant – bilateral symmetric weakness is common in the finger extensor muscles, especially predominant ulnar extensor involvement leading to claw hands, while the finger flexor, wrist extensor and wrist flexor muscles remain relatively normal. A study of 84 GBS patients reported that 12 patients with acute motor axonal neuropathy (AMAN) exhibited severe finger extensors involvement, and among them, four patients had claw hands. The author of that study believed that selective finger extensor weakness was a specific manifestation of AMAN.^[1]^ Dubey et al^[2]^ found similar symmetric claw hands in a 9-year-old child, and the electrophysiological findings were similar to those of AMAN. However, relevant publications have reported that the special variant such as selective finger extensor weakness was also found in other disease conditions besides AMAN. GBS has three subtypes defined by electrophysiological and pathogenetic findings, including acute inflammatory demyelinating polyneuropathy (AIDP), AMAN, and acute motor-sensory axonal neuropathy (AMSAN).^[8]^ In three publications, all eight patients with AIDP presented weakness in the finger extensor, of them, three patients had severe ulnar extensor involvement.^[3,4,5]^ Two other case reports showed that two patients with AMSAN also presented with predominant weakness of ulnar finger extension.^[6,7]^ Of all 23 patients described above, 8 patients with AIDP, 1 patient with AMAN, and 1 patient with AMSAN achieved a good prognosis and showed complete recovery within 6 months. However, 12 patients with AMAN had a slow recovery with persistent predominant finger extensor weakness, and 1 patient with AMSAN died from secondary respiratory paralysis. Table 2 summarizes the clinical features of these cases.

**Table 2:**
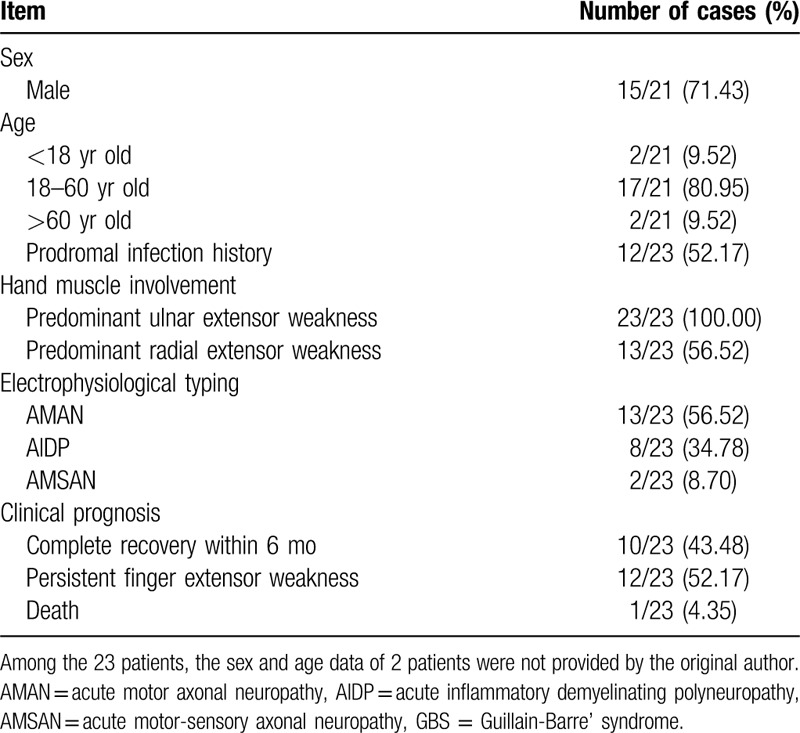
Clinical information of 23 GBS patients with hand muscle involvement.

Our report emphasizes again on the special variant of GBS with selective hand muscle involvement - finger extensor weakness. Host and environmental factors may lead to different injuries caused by compression of distal sites, which may be a precondition for development of this variant.^[2]^ In addition, the distal ulnar nerve is affected by high pressure not only from the ulnar tunnel, but also from the carpal tunnel.^[9]^ Thus, the blood-nerve barrier of the distal ulnar nerve may be more incomplete and susceptible to autoimmune attacks than the blood-nerve barrier of the distal median nerve, which is associated with predominant ulnar extensor involvement in this variant. In all relevant case reports, no specific antibody was discovered in the blood of most patients, and recovery from weakness was slow in patients with severe motor axonal damage. We speculated that this variant may involve an unknown specific antibody that plays an important role. Unlike the patients presenting with bilateral onset and rapid progression in previous reports, our patient presented with a unilateral onset and had relatively slow progression. His unilateral symptoms remained stable until six weeks later, when he developed bilateral symmetric claw hands. To date, similar cases have not been reported. We speculated that the slow progression may be related to an extension of the autoimmune response.^[10]^

In conclusion, bilateral symmetric finger extensor weakness may be a special variant of GBS with exclusive hand muscle involvement, especially the ulnar extensor involvement. Notably, it is important that GBS should also be considered in patients with unilateral hand weakness and unknown aetiology in the early stages of disease. We think that this special variant may contribute to early recognition of atypical GBS types, but a large-scale clinical observation is needed to verify the specificity and sensitivity of this variant in the future.

## Acknowledgments

The authors thank DR Na Liu of the Department of Neurology at Third Central Hospital of Tianjin for assisting in the neuronal electrophysiologic examinations.

## Author contributions

**Conceptualization:** Suhong Wang, Zhecheng Zhang.

**Data curation:** Suhong Wang, Shuxin Zhao.

**Formal analysis:** Suhong Wang.

**Investigation:** Suhong Wang, Shuxin Zhao.

**Methodology:** Suhong Wang.

**Resources:** Suhong Wang, Shuxin Zhao.

**Supervision:** Zhecheng Zhang.

**Validation:** Zhecheng Zhang.

**Visualization:** Zhecheng Zhang.

**Writing – original draft:** Suhong Wang.

**Writing – review and editing:** Suhong Wang, Zhecheng Zhang.
